# Fluorinated nanotubes: synthesis and self-assembly of cyclic peptide–poly(vinylidene fluoride) conjugates†

**DOI:** 10.1039/d1py00355k

**Published:** 2021-07-13

**Authors:** Enrique Folgado, Qiao Song, Sebastien Perrier, Vincent Ladmiral, Mona Semsarilar

**Affiliations:** Institut Charles Gerhardt Montpellier, ICGM, Univ Montpellier, CNRS, ENSCM Montpellier France; Institut Européen des Membranes, IEM, Univ Montpellier, CNRS, ENSCM Montpellier France mona.semsarilar@umontpellier.fr; Department of Chemistry, University of Warwick Gibbet Hill Road Coventry CV4 7AL UK S.Perrier@warwick.ac.uk; Faculty of Pharmacy, Monash University 381 Royal Parade Parkville VIC 3052 Australia; Warwick Medical School, The University of Warwick Coventry CV4 7AL UK

## Abstract

The synthesis of cyclic peptide–poly(vinylidene fluoride) (CP–PVDF) conjugates comprising (d-*alt*-l)-cyclopeptides as building blocks and their self-assembly into tube-like structures is described. By growing two PVDF polymeric chains from opposite sides of a preassembled cyclic-peptide macro-chain transfer agent, a PVDF–CP–PVDF conjugate was prepared. This “grafting-from” strategy, allowed the synthesis of the conjugate with high purity and using facile purification steps. The controlled self-assembly of the conjugate from DMF or DMSO solutions was carried out by addition of THF. This triggered the aggregation process that led to formation of tube-like structures. The mean length and width of the PVDF–CP–PVDF tubes were measured using atomic force microscopy (AFM) and transmission electron microscopy (TEM). Surprisingly, the self-assembly of the CP–PVDF conjugates in DMF/THF allowed the preparation of long (up to 25 μm) tube-like structures. The formation of such long tubular peptide–polymer aggregates, based on the stacking of cyclopeptides, is unprecedented and is believed to rely on synergetic effects between the stacking of the cyclic peptide and the interactions of the fluoropolymer–peptide conjugates.

## Abstract 2

Fluorinated hollow nanotubes were prepared from pre-assembled cyclic peptides bearing xanthate moieties by RAFT/MADIX of VDF.

## Graphical abstract

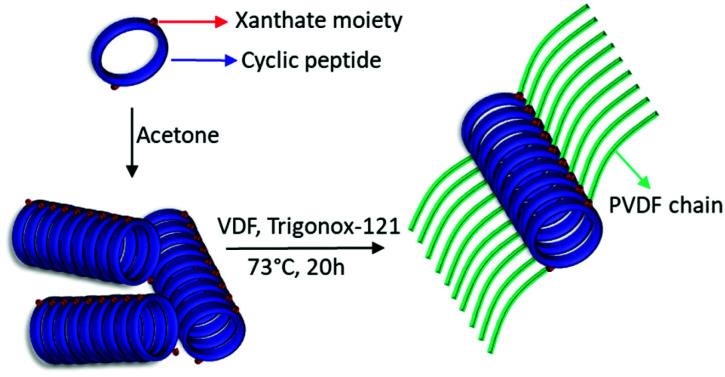

## Introduction

Recently, peptide–polymer conjugates have attracted special attention for their application in a wide range of fields, including therapeutics and separation technologies.^1–5^ A fascinating class of peptides that are known to self-assemble into supramolecular nanotubes (NTs) are cyclic peptides (CPs) comprising an even number of alternating d- and l-amino acids.^6,7^ The pioneering work on CP nanotubular structures was carried out by Ghadiri and co-workers.^6^ The alternating chirality of the amino acids in the macrocycle leads to amide bonds that alternate in orientation perpendicular to the plane of the CP rings.^8^ As a result, a contiguous intermolecular hydrogen-bonded network arises, resulting in the formation of hollow and extended cylindrical structures. In these structures, all of the amino acid side chains are directed towards the outside of the cycle. Because of this side chain arrangement, the interior of the resulting assemblies remain empty, thus creating a channel along the axis of the cyclic peptide nanotubes. In addition, both the functional groups and the diameter of the nanotube can be precisely controlled by changing the sequence and the number of amino acids in the cyclic peptide.^5,9^ Despite the great progress made with CP NTs in applications such as ion sensing,^10^ transmembrane ion channels^2,11^ and drug delivery systems,^12^ limitations with respect to their solubility, functionality and lack of control over their length restrict the expansion of applications.

Polymer conjugation allows some degree of control over the tube length, and the nature of the grafted polymer influences the solubility of the CP–polymer NTs.^13,14^ To a great extent, CP–polymer conjugates, whereby the CP has been used as a supramolecular template, have addressed these issues.^13,15–18^ Typically, CP–polymer conjugates are synthesized *via* either a grafting-from (divergent) or a grafting-to (convergent) approach.^9,19,20^ In the grafting-from approach,^3,20^ the polymer chains are grown from the peptide using a variety of polymerization techniques^21,22^ while in the grafting-to approach the polymers are synthesized separately and then grafted to the peptide using highly efficient coupling reactions.^18,23^ However, despite the use of these highly efficient reactions, such as copper(i)-catalyzed alkyne–azide cycloaddition (CuAAC),^13^ or activated ester-mediated ligations,^20^ the grafting-to approach often requires an excess of polymer and time-consuming labor-intensive purification steps to remove the unreacted polymer.^24^ In addition, the grafting-from synthetic strategy is not limited by monomer side chain functionalities orthogonal to the chain end group used for the conjugation, as in the grafting-to route.^13,24^ In recent years, many polymers have been grown from or grafted to cyclic peptides using different chemistries.^1,5,11,18,20,21,24,25^ The first report of a “grafting-from” approach was made by Couet *et al*. in 2006, who described the use of preassembled CP initiators to prepare CP–PNIPAM (poly(*N*-isopropylacrylamide)) conjugates, using surface-initiated atom transfer radical polymerization (SI-ATRP).^21^ In 2016, Larnaudie *et al.* expanded the approach to reversible addition–fragmentation chain-transfer (RAFT) polymerization, by functionalising a CP using a trithiocarbonate RAFT agent bearing an *N*-hydroxysuccinimide (NHS)moiety.^20^ They explored the polymerization of a variety of monomers, including butyl acrylate, *N*-isopropylacrylamide, *N*-acryloylmorpholine, 2-hydroxyethyl acrylate and polyethyleneglycol acrylate. They also compared the “grafting-from” and the “grafting-to” approaches and concluded that the “grafting-to” strategy is more flexible in terms of choice of solvents and polymers. However, the “grafting-from” approach affords purer conjugate in a shorter time. This approach, however, relies on the availability of a solvent that can solubilize the peptide, the monomer and the resulting conjugate at the same time.

Poly(vinylidene fluoride) (PVDF), a semi-crystalline polymer presenting excellent physicochemical properties as well as electroactive properties (piezoelectricity, pyroelectricity and ferroelectricity) is used in very diverse fields.^26–31^ In addition to its electroactive properties, PVDF is also often use in filtration membranes. CP–PVDF conjugates self-assembled nanotubes could offer a way to design and fabricate PVDF-based membranes endowed with well-defined functionalized channels.

**Scheme 1:**
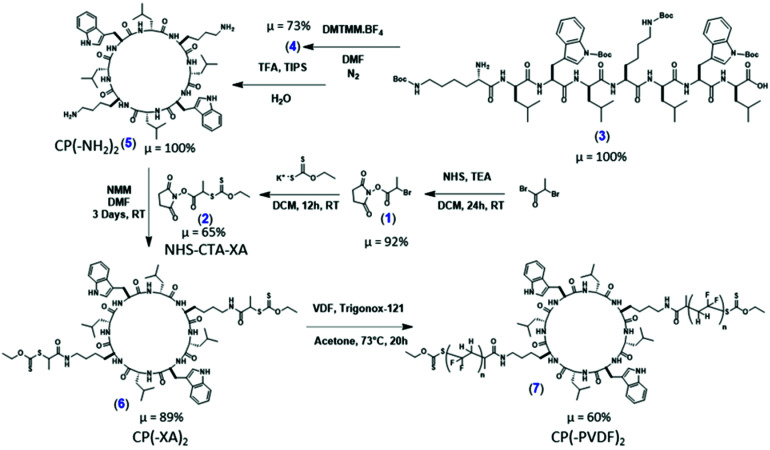
Synthetic route to the cyclic peptide–PVDF conjugate employing a “grafting-from” approach.

We report herein the fabrication of the first CP–PVDF conjugates, as illustrated in Scheme 1, constructed *via* a divergent synthetic approach using a CP(-Xanthate)_2_ building block *via* Macromolecular Architecture Design by Interchange of Xanthates (MADIX). In appropriate solvents, self-assembly is possible, resulting in well-defined PVDF nanotubular structures. The use of a CP containing eight alternating d- and l-amino acids permits a facile templated approach for the formation of well-defined PVDF NTs featuring sub nanometer channels within their cores.

## Experimental section

### Materials

*N*-Hydroxysuccinimide (98%), triethylamine (TEA) (>99.5%), 2-bromopropionyl bromide (97%), magnesium sulfate anhydrous (>99.5%), potassium ethyl xanthogenate (96%), 4-dimethylaminopyridine (DMAP, 99%), 2-chloro-4,6-dimethoxy-1,3,5-triazine (97%), triisopropylsilane (TIPS, 99%), and aluminum oxide were purchased from Sigma-Aldrich. 1,1,1,3,3,3-Hexafluoroisopropanol (HFIP, 99%) and iodine were purchased from Acros Organics. *N*-Methylmorpholine (NMM, 99%) and piperidine were purchased from Alfa Aesar. Sodium hydroxide pellets, sodium thiosulfate pentahydrate and anhydrous magnesium sulfate (MgSO_4_) were purchased from Fisher. *N*,*N*-diisopropylethylamine (DIPEA, 99%), was purchased from Merck. Fmoc-d-Leu-OH, Fmoc-l-Lys(Boc)-OH, Fmoc-l-Trp(Boc)-OH, *O*-(1*H*-6-chlorobenzotriazole-1-yl)-1,1,3,3-tetramethyluronium hexafluorophosphate (HCTU) and 2-chlorotrityl chloride resin (100–200 mesh) were purchased from Iris Biotech and used as received. *tert*-Amyl peroxy-2-ethylhexanoate (Trigonox 121, purity 95%) was purchased from AkzoNobel (Chalons en Champagne, France). Deuterated solvents for NMR were purchased from Euristop. All solvents were bought from commercial sources and used as received. VDF was kindly supplied by ARKEMA. The cyclization coupling agent 4-(4,6-dimethoxy-1,3,5-triazin-2-yl)-4-methylmorpholinium tetrafluoroborate (DMTMM·BF4) was synthesized according to an established literature method.^8^

### Nuclear magnetic resonance (NMR)

The NMR spectra were recorded on a Bruker AV III HD Spectrometer (400 MHz for ^1^H and 376 MHz for ^19^F). Coupling constants and chemical shifts are given in hertz (Hz) and parts per million (ppm), respectively. The experimental conditions for recording ^1^H and ^19^F NMR spectra were as follows: flip angle, 30°; acquisition time, 4 s; pulse delay, 1 s; number of scans, 16 (or 32 for ^19^F); and pulse widths of 9.25 and 11.4 μs for ^1^H and ^19^F NMR respectively.

### Dynamic light scattering (DLS)

DLS measurements of polymer solutions were carried out with an Anton Paar Litesizer™ 500 using a quartz cuvette at 25 °C.

### Transmission electron microscopy (TEM)

TEM studies were conducted using a JEOL 1400+ instrument equipped with a numerical camera, operating with a 120 kV acceleration voltage at 25 °C. To prepare TEM samples, a drop (10.0 μL) of micellar solution was placed onto a Formvar/carbon or Lacey/carbon coated copper grid for 30 s, blotted with filter paper and dried under ambient conditions.

### Scanning electron microscopy (SEM)

SEM analyses were conducted using a Hitachi S-4500 instrument operating at spatial resolution of 1.50 nm at 15 kV energy. The samples were prepared by spin coating of 50 μL of the solution on a silicon wafer. The samples were then placed on a flat mount after being coated with an ultrathin layer of electrically conducting platinum deposited by high-vacuum evaporation.

### Atomic force microscopy (AFM)

AFM samples were prepared by spin coating of 50 μL (diluted 10 times in acetone in the case of PVDF-CP crude of polymerization reaction) of the solution on a freshly cleaved mica wafer. AFM images were acquired with a Pico SPM II provided by Molecular Imaging. The imagery was controlled by the PicoView 1.10 software. The experiments were all carried out in tapping mode. The types of tips used were PPS-FMR purchased from Nanosensors with a frequency resonance between 45 and 115 kHz and a force constant between 0.5 and 9.5 N m^−1^. Gwyddion 2.25 software was used to treat the images. Samples were prepared by spin coating 50 μL of the solution on the surface of freshly cleaved mica wafers (sample concentration 0.1 mg mL^−1^ in DMF : THF (1 : 9)).

### Mass spectrometry

Measurements were performed on a Bruker MicroToF for ESI ToF and on an Agilent 6130B Single Quad for ESI.

### Gel permeation chromatography (GPC)

GPC was measured using an Agilent PL50 instrument with a differential refractive index detector. The instrument contained two PolarGel H columns (300 mm × 7.5 mm) and a PolarGel 5 μm guard column. DMF with 0.1% LiBr additive was used as the eluent. The system ran at 1 mL min^−1^ (50 °C), with an injection volume of 100 μL. The samples were prepared by filtering them through 0.22 μm pore size PTFE membranes, before injection. Agilent EasyVial poly(methyl methacrylate) standards were used to calibrate the instrument and output data were analyzed using Agilent GPC/SEC software.

### Synthesis

#### Synthesis of *N*-succinimidyl bromoacetate, (Scheme 1, 1)

*N*-Hydrosuccinimide (NHS) (6.33 g; 55 mmol) was placed in a 250 mL round bottom flask and dissolved in 80 mL of DCM under magnetic stirring. The flask was placed in an ice bath and TEA (8.1 mL, 58 mmol) in 16 mL of DCM was added dropwise. After stirring for 30 min, 2-bromopropionyl bromide (6.08 mL; 58 mmol) in 16 mL of DCM was added dropwise over a period of 1 h. The reaction was left for 24 h at room temperature (25 °C). The mixture was then washed with brine and the organic phase was collected and dried over anhydrous magnesium sulfate. The solvent was removed by rotary evaporation. The brownish solid was dissolved in isopropanol at 75 °C and a few drops of DCM were added. The product was left to crystallize in the fridge and was filtered to yield 92%.

#### Synthesis of NHS-CTA-XA, (Scheme 1, 2)

*N*-Succinimidyl bromoacetate (6 g; 24 mmol) was placed in a 100 mL round bottom flask and dissolved in 45 mL of absolute ethanol. The flask was placed in an ice bath and potassium ethyl xanthogenate (4.8 g; 29 mmol) was added with a spatula over a period of 45 min. The heterogeneous solution was stirred 3 h at room temperature, then filtered over Celite and the solvent removed by rotary evaporation. The product was dissolved in 90 mL of DCM and washed with pure water (4 × 150 mL) and dried over magnesium sulphate then solvent was removed by rotary evaporation yielding a crystalline yellow powder (65%).

#### Synthesis of the cyclic peptide CP(–NH_2_)_2_

Standard Fmoc-deprotection solid-phase peptide synthesis was used to first synthesize the protected linear peptide. Using a coupling agent, the cyclization was completed in dilute conditions to avoid intermolecular reactions. The insolubility of the stacked cyclic peptides in methanol was used to isolate the pure cyclic peptide. The cyclic peptide was then deprotected using TFA to reveal the amines of the lysine residues and azoles on the tryptophan residues.^20^

#### Synthesis of the linear peptide H2N-l-Lys(Boc)-d-Leu-l-Trp(Boc)-d-Leu-l-Lys(Boc)-d-Leu-l-Trp(Boc)-d-Leu-COOH, (Scheme 1, 3)

Fully protected linear octapeptide was prepared *via* solid phase peptide synthesis (SPPS) on a Prelude Automated Peptide Synthesizer™ (Protein Technologies Inc.) using 2-chlorotrityl chloride resin as the solid support. The first Fmoc-protected amino acid was coupled to the resin using DIPEA (4 eq.) in DMF, followed by capping of unreacted resin sites using a solution of MeOH : DIPEA : DCM (7 : 1 : 2, v/v/v). Deprotection of the Fmoc group of the amino acids was done using 20% piperidine in DMF. Subsequent amino acids were coupled using Fmoc-amino acids (5 eq.), HCTU (5 eq.) and NMM (10 eq.) in DMF. In the last step, the linear octapeptide was cleaved from the resin (while keeping protecting groups on) by a solution of 20 vol% 1,1,1,3,3,3-hexafluoro-2-propanol (HFIP) in DCM. Yield quantitative.

#### Protected cyclic peptide cyclo(-l-Lys(Boc)-d-Leu-l-Trp(Boc)-d-Leu-l-Lys(Boc)-d-Leu-l-Trp(Boc)-d-Leu-), (Scheme 1, 4)

Linear peptide (200 mg, 0.127 mmol) was dissolved in DMF (20 mL) and N_2_ was bubbled through the solution for 20 min. DMTMM·BF_4_ (1.2 eq., 51 mg, 0.152 mmol) was dissolved in DMF (5 mL), N_2_ bubbled through the solution for 20 min, then this solution was added dropwise to the linear peptide solution. The mixture was stirred under an atmosphere of N_2_ for 5 days. The DMF solution was reduced to a volume of ∼1 mL under reduced pressure, and methanol (20 mL) was added. Aliquots of the suspension were distributed into 2 mL Eppendorf tubes and centrifuged at 10 000 rpm for 4 minutes using a benchtop centrifuge. After removal of the supernatant, the pellets were redispersed in methanol. The Eppendorf tubes were centrifuged once more and the supernatant discarded. The pellets were redispersed in methanol and the solvent was evaporated under reduced pressure to yield the Boc-protected cyclic peptide in the form of a white powder. Yield 73% (138 mg, 0.093 mmol). MS (ESI) [M + Na]^+^ calculated: 1503.89, found: 1503.8.

#### Deprotected cyclic peptide cyclo(-l-Lys-d-Leu-l-Trp-d-Leu-l-Lys-d-Leu-l-Trp-d-Leu-), (Scheme 1, 5)

Boc groups were removed using a deprotection solution of TFA/TIPS/H_2_O (18 : 1 : 1 vol, 5 mL). The protected cyclic peptide was stirred for 2 hours in the deprotection solution, then precipitated using chilled diethyl ether and washed twice more with chilled diethyl ether. The off-white powder was collected and dried under vacuum. Yield: quantitative.

#### Synthesis of the cyclic peptide chain transfer agent, (Scheme 1, 6)

The desired cyclic peptide chain transfer agent CP-(XA)_2_ was obtained by coupling the chain transfer agent (NHS-CTA-XA) to the lysine residues of CP (Scheme 1). The CP (120 mg; 0.11 mmol; 1 eq.) was dissolved in 6 mL DMSO. Complete dissolution was reached after 10 min in ultrasound bath. Then NHS-CTA-XA (64.68 mg; 0.222 mmol, 2 eq.) and NMM (0.074 mL; 0.666 mmol, 3eq.) were added and the solution was stirred at room temperature for 3 days. Mass spectrometry monitoring indicated that the coupling reaction was quantitative, as no residual CP or mono-functionalized product was detected, affording CP-(XA)_2_ in high yield (89%). The product was precipitated twice in chilled diethyl ether and dried under vacuum. MS (ESI) (Fig. S4†) [M + Na]^+^ calculated: 1433.91, found: 1433.95.

#### VDF RAFT/MADIX polymerization using NHS-CTA-XA in acetone

RAFT polymerization was carried out in a thick Carius tube containing NHS-CTA-XA (38 mg, 13.0 × 10^−2^ mmol), acetone (7 ml) and the initiator (Trigonox-121) (3.3 mg, 13.0 × 10^−1^ mmol) were mixed and the tube was degassed with three freeze–pump–thaw cycles to remove any trace of oxygen. The gaseous VDF monomer (0.5 g, 7.81 mmol) was transferred into the Carius tube and cooled in liquid nitrogen. The tube was then sealed, before being placed horizontally in a shaking water bath thermostated at 73 °C. After 24 hours, the tube was frozen in liquid nitrogen, opened and left to return to ambient temperature. Once room temperature was reached, the crude sample was precipitated twice in a tenfold excess of chilled pentane. The NHS-PVDF polymer was recovered by centrifugation at 4000 rpm for 15 min in 10 mL conical centrifuge tubes. The polymer was dried overnight under vacuum at 25 °C (Yield = 60%). Yield was used as conversion since conversion is very difficult to calculate accurately for gaseous monomers.

#### VDF RAFT/MADIX grafting-from polymerization using CP-(XA)_2_, (Scheme 1, 7)

RAFT polymerization was carried following the same protocol described above. CP-(XA)_2_ (93 mg, 6.51 × 10^−2^ mmol), acetone (7 ml) and the initiator (Trigonox-121) (3 mg, 13.0 × 10^−1^ mmol) were sonicated for 10 min or until complete CP-(XA)_2_ dispersion before degassing and introducing the gaseous VDF monomer (0.5 g, 7.81 mmol). Conversion was not estimated since the study of the aggregates in solution required no further purification. However, by weighing the Carius tube before and after polymerization (after breaking the glass tube to allow unreacted VDF to evaporate) a yield of 50% was estimated.

### Preparation of CP–(PVDF)_2_ solutions

Stock solutions of 1 mg mL^−1^ of CP–(PVDF)_2_ were prepared in DMF or DMSO, using ultrasounds and heating at 60 °C until full solubilisation.

### Preparation of the self-assembled nanotubes

0.1 mL of the solutions described above were placed in stirring plates with magnetic stirring bars. Then, 0.9 or 9.9 mL of THF were added dropwise using a syringe pump at a fixed rate of (4 mL h^−1^) (final conjugate concentration and solvent ratios of 0.1 or 0.01 mg mL^−1^ and 1 : 9 or 1 : 99 respectively). The solutions were let stirred slowly for 24 h. 10 μL and 50 μL were taken to prepare TEM and AFM samples respectively.

## Results and discussion

### Synthesis of the cyclic peptide chain transfer agent

The initial cyclic peptide (CP(-NH_2_)_2_) (sequence of amino acids: l-Lys-d-Leu-l-Trp-d-Leu-l-Lys-d-Leu-l-Trp-d-Leu) (see Scheme 1) was synthesized according to previously reported procedures.^20^ The desired cyclic peptide chain transfer agent CP-(XA)_2_ was obtained by coupling the chain transfer agent (NHS-CTA-XA) to the lysine residues of the CP. Mass spectrometry indicated that the reaction proceeded quantitatively in 72 hours. Although aminolysis of the xanthate moiety by the lysine residues is a potential side reaction, no evidence of such corresponding *O*-thiocarbamate formation was found.

### Study of the suitable polymerization conditions

Recent studies show that xanthate RAFT agents, peroxide initiators and dimethyl carbonate (DMC) as solvent are the best conditions for the RAFT/MADIX polymerization of VDF.^32^ VDF polymerization in DMC proceeds faster than in other organic solvents, affording high yields, and also leads to relatively small quantities of –CH_2_–CF_2_–H terminated dead chains (mainly formed by radical transfer from –CF_2_˙ radicals to DMC).^32,33^ Unfortunately, the prepared CP(-XA)_2_ is not soluble in DMC therefore preventing its use as solvent. Whilst DMF and DMSO are both suitable solvents for the CP and PVDF and the solvent of choice for most polymerizations initiated from CP macro CTA,^20,22^ both solvents act as chain transfer agents in the polymerization of VDF, thus leading to poor conversion.^33^ We therefore turned our attention to an alternative solvent as compromise, acetone, which is a suitable solvent for the polymerization of VDF, although more prone to H-abstraction than DMC.^32^ While the unmodified CP was insoluble in acetone, the modified CP macroCTA (CP(-XA)_2_) formed a stable dispersion in acetone. This milky appearance of the solution was attributed to the strong tendency of the CP to self-assemble into nanotubes (see S20†). A test polymerization of VDF using NHS-CTA-XA as RAFT agent was performed in acetone to assess the control of the polymerization. ^1^H NMR spectroscopy confirmed that PVDF was formed albeit at the cost of loss of end-group functionality, due to the expected increase in transfer events to the solvent (*i.e.*, higher amount of dead chains: –CH_2_–CF_2_–H and –CF_2_–CH_3_, signals at 6.3 and 1.7 ppm (Fig. S5†), −92.00, −114.5 and −107.7 ppm (Fig. S6†) respectively). The molar fractions of the different end-groups were determined using eqn (S3) to (S6)† and data from ^19^F NMR spectrum (Fig. S8†), estimated to be: –CF_2_-XA (0%), –CH_2_-XA (40.4%), –CF_2_–CH_3_ (6.2%) and –CF_2_H (49.4%). It is noteworthy that in comparison, a typical VDF polymerization in DMC leads to up to 15% of –CF_2_H and 85% of –CH_2_-XA for a polymer of DP = 50,^33^ although irreversible transfer reactions tend to prevail at high conversions. Thus, higher DP with high functionality can only be obtained at low conversions due to a progressive loss of xanthate groups.^34^ It is therefore clear that the RAFT polymerization of VDF does do not follow the typical molecular weight control and kinetics expected from a RAFT system. The conditions used here are however, a compromise based on the solubility of the CTA, and provide a PVDF that retains its α-chain end group, attached to the cyclic peptide. These results suggests that the grafting-from RAFT polymerization of VDF using acetone as solvent is a good compromise if low DPs are targeted and the xanthate ω-end group functionality can be sacrificed.

### Synthesis of the CP–PVDF conjugate *via* “grafting-from” RAFT polymerization of VDF

The RAFT polymerization of VDF was carried out in the milky suspension of CP(-XA)_2_ in acetone. This system is presumably more akin to a grafting-from system due to the suboptimal solubility of the macroCTA in acetone. Visually, no changes were observed in terms of solubility or colloidal stability at the end of the polymerization.

**Fig. 1:**
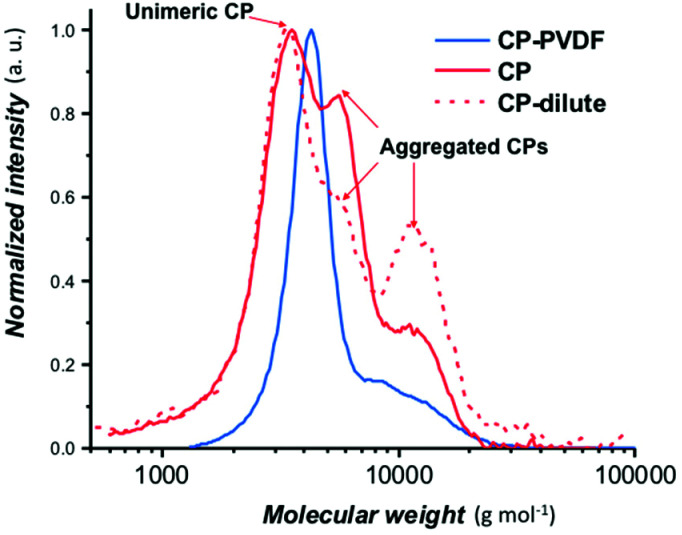
Normalized SEC chromatograms (RI detector) of a concentrated cyclic peptide (CP; red trace), a diluted cyclic peptide solution in DMF (CP; red discontinuous trace) and a solution of CP–(PVDF)_2_ (CP–PVDF; blue trace).

The GPC trace (Fig. 1) of CP(PVDF)_2_ suggests that all the PVDF chains have grown from the CP(CTA)_2_ since no shoulders are observed at lower molar masses. The dispersity of this CP(PVDF)_2_ (1.26) was comparable to the typical dispersity of PVDF obtained by RAFT polymerization.^32,34^

The PVDF segments grown from the CP appear to have an average DP of 45 from NMR calculations (using eqn (S1)†), higher than the estimated theoretical DP of 30 (calculated from eqn (S1), see ESI†). The results suggest that due to the partial solubility of the CP(XA)_2_ macroCTA in acetone, all the xanthate sites were not available for VDF polymerization leading to longer PVDF chains.

Interestingly, this polymerization also led to PVDF chains possessing large amounts of regular end-groups (–CH_2_CF_2_-XA). The molar fractions of the end-groups determined from the ^19^F NMR spectrum (Fig. S8†) were found to be –CF_2_-XA (36.0%), –CH_2_-XA (9.7%), –CF_2_–CH_3_ (8.2%) and –CF_2_H (46.1%). These values are surprising as previous studies, including the RAFT polymerization conducted here using NHS-CTA-XA, had shown that the RAFT polymerization of VDF quickly leads to the accumulation of the reversely terminated end-groups (–CF_2_–CH_2_-XA) due to their lower reactivity towards the majority radicals –CF_2_˙.^32,33,35^ In any case, –CF_2_-XA chains were not present at an advanced polymerization stage.^32,34^

### Self-assembly

**Fig. 2:**
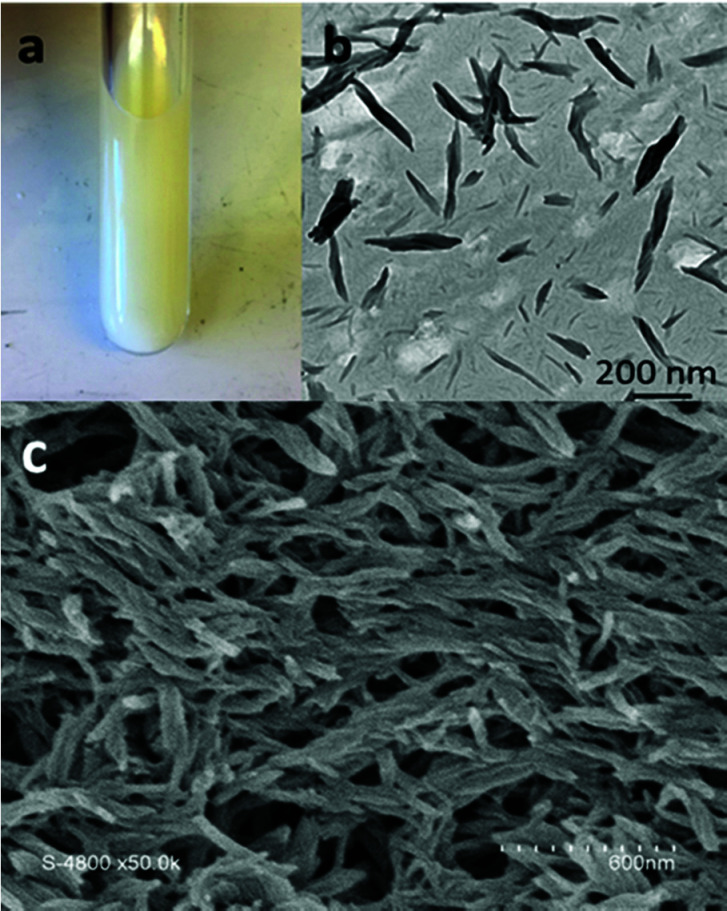
(a) Photograph of the Carius tube containing the acetone suspension after polymerization of VDF in the presence of CP-(XA)_2_, (b) TEM image of the diluted suspension shown in (a), (c) SEM image of thin films prepared from the crude CP–(PVDF)_2_ conjugate (without further purification or dilution) by spin coating of 50 μL of suspension on a silicon wafer.

The alternating d- and l-amino acid conformation of the peptide leads to the stacking of the CP *via* H-bonds perpendicular to the plane of the CP ring between the amide groups to form cylindrical structures.^6,36,37^ The l-lysines positioned on the opposite sides of the peptide ring provide useful primary amines to anchor functional groups such as the xanthates chain transfer agent used here. Initially, the crude product resulting from the polymerization of VDF was examined by electron microscopy to observe the structures present in the acetone suspension (Fig. 2a).

TEM images (Fig. 2b) show that the CP–(PVDF)_2_ spontaneously self-assembled in acetone to form aggregates of various sizes that resemble short rods and twisted ribbons (Fig. 2b and c and S11†). The width of these objects was not homogeneous (30 to 100 nm) and exceeded, in most cases, the diameter of the CP–(PVDF)_2_. These aggregates were stable even at 50 °C and after sonication (Fig. S10†).

These PVDF-based tubular aggregates can be directly deposited on a substrate by spin-coating creating a porous network (Fig. 2c). Such material might be interesting for the preparation of thin-film membranes.

**Fig. 3:**
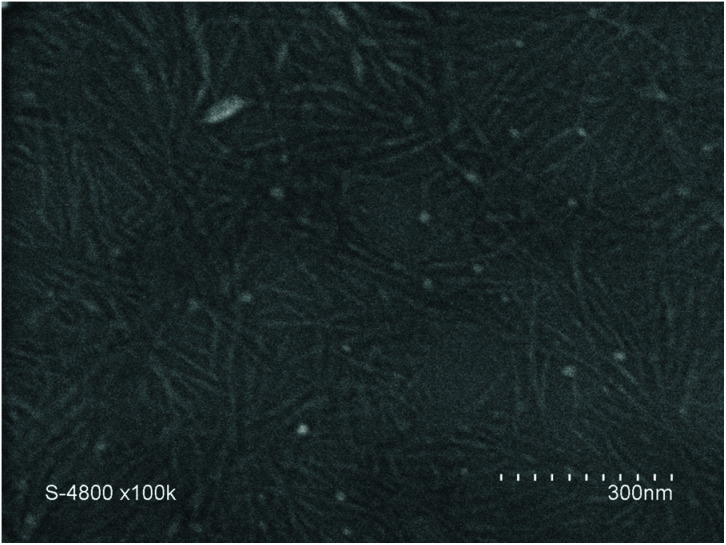
SEM image of CP(–PVDF)_2_ prepared from a 1 mg mL^−1^ DMF solution by adding THF (addition rate = 4 mL h^−1^). Final concentration 0.1 mg mL^−1^ (DMF 10% v/v in the final solution).

**Fig. 4:**
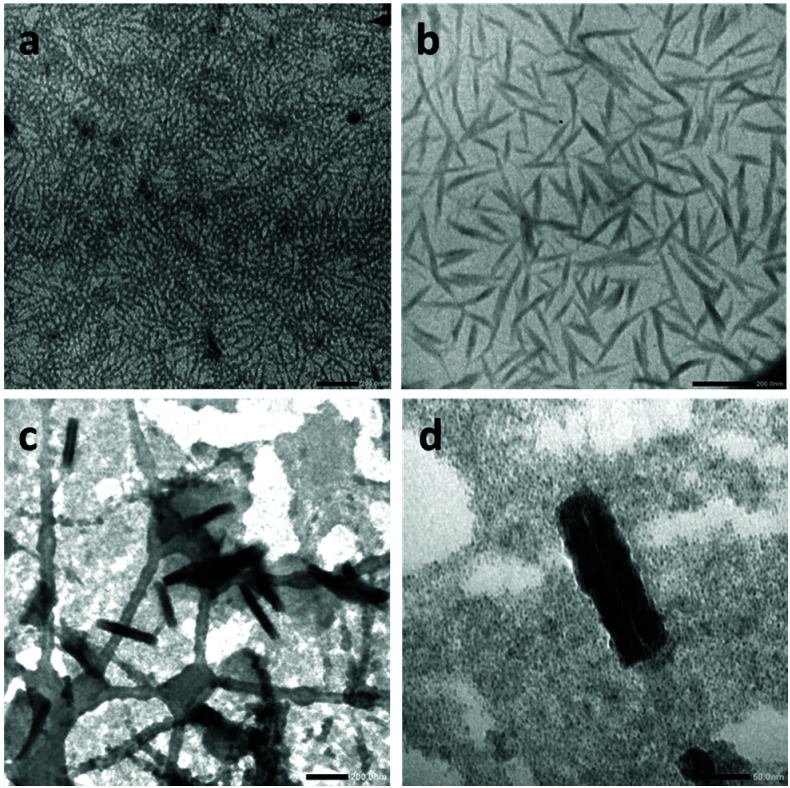
TEM image of CP(-PVDF)_2_ prepared from a 1 mg mL^−1^ DMF solution by adding THF (addition rate was 4 mL h^−1^). Final concentration (a and b) 0.1 mg mL^−1^ and (c and d) 0.01 mg mL^−1^ (DMF 10 and 1% v/v respectively in the final solutions). Sample prepared in Formvar/carbon coated copper TEM grid (a and b). Sample prepared in Lacey/carbon coated copper TEM grid (c and d) scales bars are 200 nm (a, b, and c) and 50 nm (d).

To study the self-assembly properties of the CP–(PVDF)_2_ conjugates, we attempted to first disassemble these assemblies, and then to re-assemble them in a controlled manner. The disassembly was investigated by dynamic light scattering (DLS) in presence of solvents that are known to disrupt H-bonds between the CP, trifluoroacetic acid (TFA), dimethyl-sulfoxide (DMSO) and *N*,*N*-dimethylformamide (DMF), which are also good solvents for PVDF.^2,13,16,37^ However, solutions of CP–(PVDF)_2_ in TFA turned black within 1 h, probably due to the degradation of the CP moieties. Light scattering measurements of the DMF and DMSO solutions of the CP–(PVDF)_2_ conjugates at room temperature suggested the presence of large aggregates, in contrast to what is typically observed for other types of polymer–CP conjugates. The crystallinity of PVDF likely prevented complete disassembly of the conjugates, despite the disruption of H-bonds between CPs. To disrupt this crystallinity-driven assembly, the solutions were heated to 60 °C (in DMF) and 80 °C (in DMSO) for 1 h and sonicated for 20 min. Under these conditions, DLS analyses indicated complete dissolution of the aggregates (Fig. S12†). The controlled re-assembly of the CP–(PVDF)_2_ conjugates was then carried out by slow addition, to these solutions, of THF (a good solvent of low molar mass PVDF). This procedure help to limit the disruption of H-bonds by solvent and promote assembly between CPs.^18^ In the THF/DMF solution, the CP(-PVDF)_2_ self-assembled into cylindrical aggregates as observed by SEM (Fig. 3), which revealed structures with lengths up to 250 nm. TEM analyses were also carried out (Fig. 4), to show a more detailed structure of the aggregates.

**Fig. 5:**
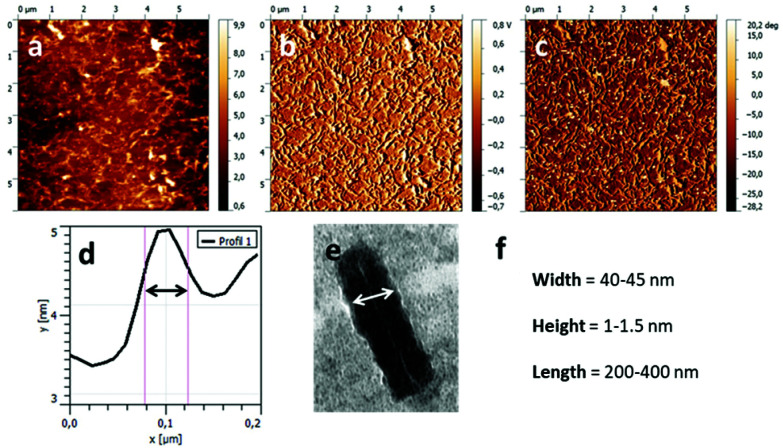
AFM images of the nanotubes formed by CP(-PVDF)_2_ deposited onto mica substrate. The sample was prepared from a 1 : 9 DMF : THF solution (conjugate concentration 0.1 mg mL^−1^). (a) Topography, (b) amplitude, (c) phase, (d) height profile and width measurement, (e) width from TEM image, (f) values extracted from the analysis TEM and AFM images.

Initial analyses showed a high concentration of cylindrical structures (Fig. 4a and b), which were further imaged after diluting the solution ten times with pure THF and depositing the solution on a Lacey/carbon TEM grid (Fig. 4c and d). This approach resulted in better contrast and higher resolution, and revealed that the cylindrical structures were in fact nanotubes (with length and diameter ranging from 200 to 400 nm (see Fig. S13†) and from 30–40 nm respectively), which inner channel can be seen on Fig. 5d.

Further study of the self-assembled objects was performed using AFM (Fig. 5), after spin-coating a solution of conjugates onto a mica substrate. Fig. 5a–c shows an extended area of fiber like aggregates, which length and diameter, extracted from the topographic image (Fig. 5a) compare well with the data obtained from TEM images. The obtained average results (Fig. 5f) were used to determine the aggregation number (*N*_agg_, number of stacked CPs). Considering the distance between each CP, reported to be 0.47 nm (ref. 25) and the data extracted from the TEM and AFM (length = 200–300 nm), *N*_agg_ was estimated between 425 and 638. The width of these aggregates is dictated by the CP diameter and the DP of PVDF. PVDF likely crystallized in its α-phase. In such a crystal PVDF segments of 45 units are estimated to have an approximate length of 9 nm.^38^ The CP diameter is about 1 nm (with an internal diameter of 0.7–0.8 nm). These estimations lead to a CP(-PVDF)_2_ conjugate of *ca.* 19 nm, which is smaller than the width observed by TEM images (40–45 nm). This discrepancy may be explained by analytical errors on the resolution from the TEM measurements in this size range and on the DP of the PVDF that may be underestimated if not all the xanthate moieties generated a PVDF chain.

**Fig. 6:**
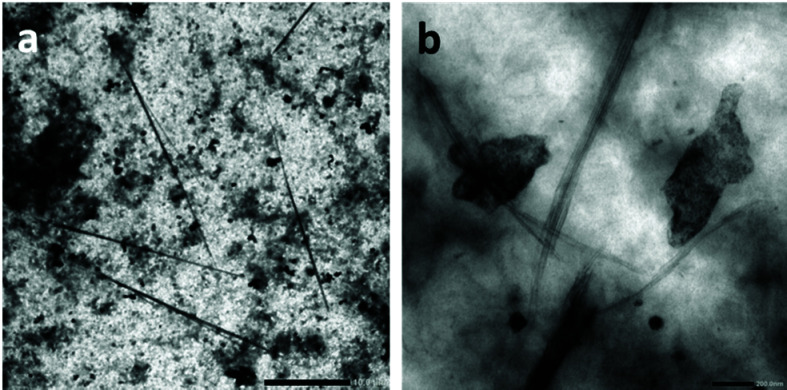
SEM image of CP(-PVDF)_2_ prepared from a 1 mg mL^−1^ DMF solution by adding THF (addition rate was 4 mL h^−1^). Final concentration 0.1 mg mL^−1^ (DMF 10% v/v in the final solution). Scale bars are 10 μm (a) and 200 nm (b).

The assemblies formed from the CP(-PVDF)_2_ solution in DMSO (Fig. 6) revealed to be much longer cylindrical structures, of up to 25 μm in length (Fig. 6a) which appeared to be composed of laterally aggregated peptide–polymer nanotube (Fig. 6b).

Bundles of laterally stacked peptides in deuterated DMSO have been reported before.^14^ Koh *et al.* observed bundles of short tubes from unconjugated CPs stacked laterally in deuterated DMSO.^14^ This type of assemblies was also observed in the case of partially conjugated CP–polymer with polybutylacrylate (PBA), due to the poor compatibility between PBA and DMSO.^14^ We hypothesize that since some of the polymerization sites were not accessed during the VDF polymerization in the acetone milky suspension of CP(XA)_2_, the resulting partially conjugated CPs may laterally stacked into the observed tubes, similar to what was observed in the case of CP–PBA.

The lengths observed by TEM (25 μm) suggest a *N*_agg_ of up to 53 200, which is greater than any values reported for similar cyclic peptide systems. Such long assemblies have not been reported for other CP–polymer conjugates, suggesting that PVDF polymer chains have an important effect on the assembly of these conjugates.

## Conclusions

The synthesis of a cyclic peptide macroCTA bearing two xanthate moieties (CP(-XA)_2_) was successfully achieved by the coupling of a NHS-functionalized RAFT agent onto the lysine residues of the CP. The RAFT/MADIX polymerisation of VDF in the presence of this CP(-XA)_2_ difunctional macroCTAs was carried out in acetone, although CP(-XA)_2_ formed a stable suspension in this solvent rather than a solution. The success of the polymerisation was however confirmed by ^19^F NMR. The signal of the first VDF unit directly connected to the R-group of the macroCTA confirmed the growth of PVDF from the CP macroCTA. Also, the presence of VDF-XA signals confirms that the polymerisation was controlled by a RAFT mechanism. Nevertheless, these polymerization conditions led to a significant loss of xanthate termini due to a high extent of transfer (three times higher than in DMC). After its disassembly in DMSO or DMF, the formation of tubular structures of different lengths was triggered using THF as the PVDF-selective solvent. These PVDF-based tubular aggregates might also find applications in separation and membrane science due to the porosity brought by the CP nanotubes.

## Author contributions

The manuscript was written through contributions of all authors./All authors have given approval to the final version of the manuscript.

## Funding sources

The authors thank Institut Carnot Chimie Balard Cirimat, LabEx CheMISyst (ANR-10-LABX-05-01), IEM, ICGM (E. F.; V. L.; M. S.), CNRS-Osez l'Interdisciplinarité 2017 (MS), Monash-Warwick Alliance (Q. S.; S. P.), European Research Council (TUSUPO 647106; Q. S.; S. P.). S. P. and M. S. also thank the Royal Society for financial support (Royal Society International Exchanges grant IE161841).

## Conflicts of interest

There are no conflicts to declare.

## Supplementary Material

PY-012-D1PY00355K-s001
