# Supplementary material for: Fluorinated nanotubes: synthesis and self-assembly of cyclic peptide–poly(vinylidene fluoride) conjugates
Source: Polym Chem. 2021 Jul 13;12(29):4235–43. doi: 10.1039/d1py00355k (PMC8734003; doi:10.1039/d1py00355k)
Supplement: PY-012-D1PY00355K-s001 [file PY-012-D1PY00355K-s001.pdf]

Electronic Supplementary Material (ESI) for Polymer Chemistry.
This journal is © The Royal Society of Chemistry 2021

Supporting information

Fluorinated Nanotubes: Synthesis and self-assembly of cyclic peptide- poly(vinylidene fluoride) conjugates

Enrique Folgado,[a,b,‡] Qiao Song,[c,‡] Sebastien Perrier,[c,d,e*] Vincent Ladmiral,[a*] Mona Semsarilar[b*]

[a]Institut Charles Gerhardt Montpellier, ICGM, Univ Montpellier, CNRS, ENSCM, Montpellier, France.

[b]Institut Européen des Membranes, IEM, Univ Montpellier, CNRS, ENSCM, Montpellier, France.

[c]Department of Chemistry, University of Warwick, Gibbet Hill Road, Coventry CV4 7AL, UK.

[d]Faculty of Pharmacy, Monash University, 381 Royal Parade, Parkville, VIC 3052, Australia

[e]Warwick Medical School, The University of Warwick, Coventry CV4 7AL, UK.

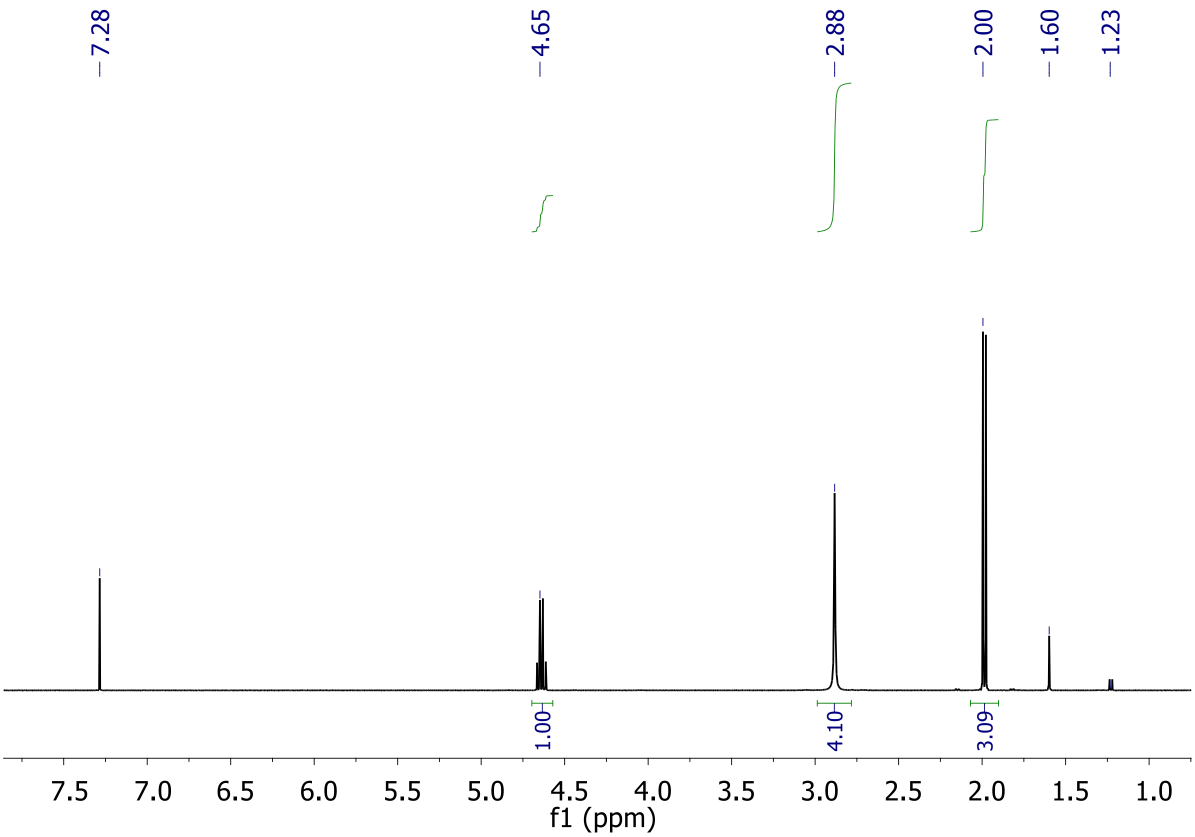

^{1}H NMR (400 MHz, $CDCl_3$, ppm, Figure S1) δ = 4.65 (q, J = 7.4 Hz, 1H), 2.88 (s, 4H), 2.00 (d, J = 7.4 Hz, 3H)

Figure S1. ^{1}H NMR spectrum ($CDCl_3$, 400 MHz) of *N*-succinimidyl bromoacetate.

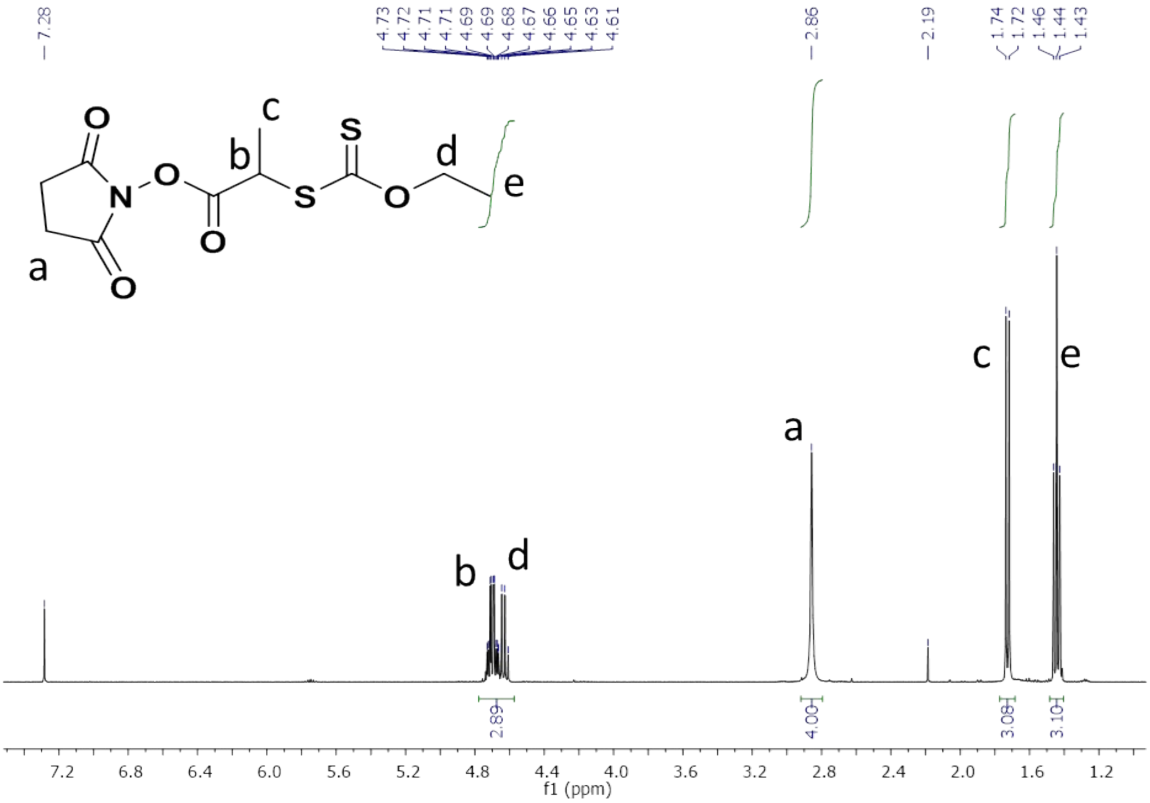

^{1}H NMR (400 MHz, $CDCl_3$, ppm, Figure S2) δ = 4.70 (dq, J = 7.1, 2.3 Hz, 1H), 4.64 (q, J = 7.4 Hz, 2H), 2.86 (s, 4H), 1.73 (d, J = 7.4 Hz, 3H), 1.44 (t, J = 7.1 Hz, 3H).

Figure S2. ^{1}H NMR spectrum ($CDCl_3$, 400 MHz) of NHS-CTA-XA.

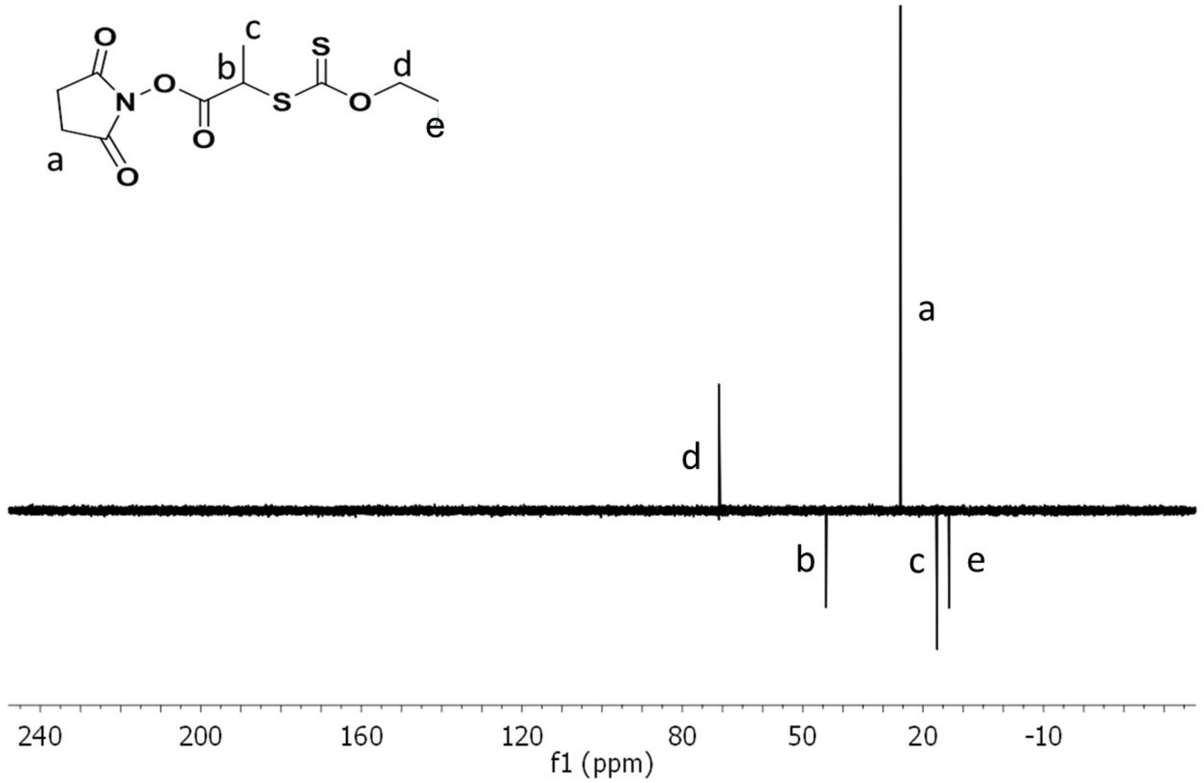

^{13}C {1H} DEPT-135 NMR (100 MHz, $CDCl_3$, Figure S3) δ = 13.52 (-O-CH_2-CH_3), 16.50 (-CH(CH_3), 25.60 (-CH_2-CH_2-), 44.19 (-CH(CH_3), 70.91 (-O-CH_2-CH_3).

Figure S3. ^{13}C DEPT 135 NMR spectrum ($CDCl_3$, 101 MHz) of NHS-CTA-XA.

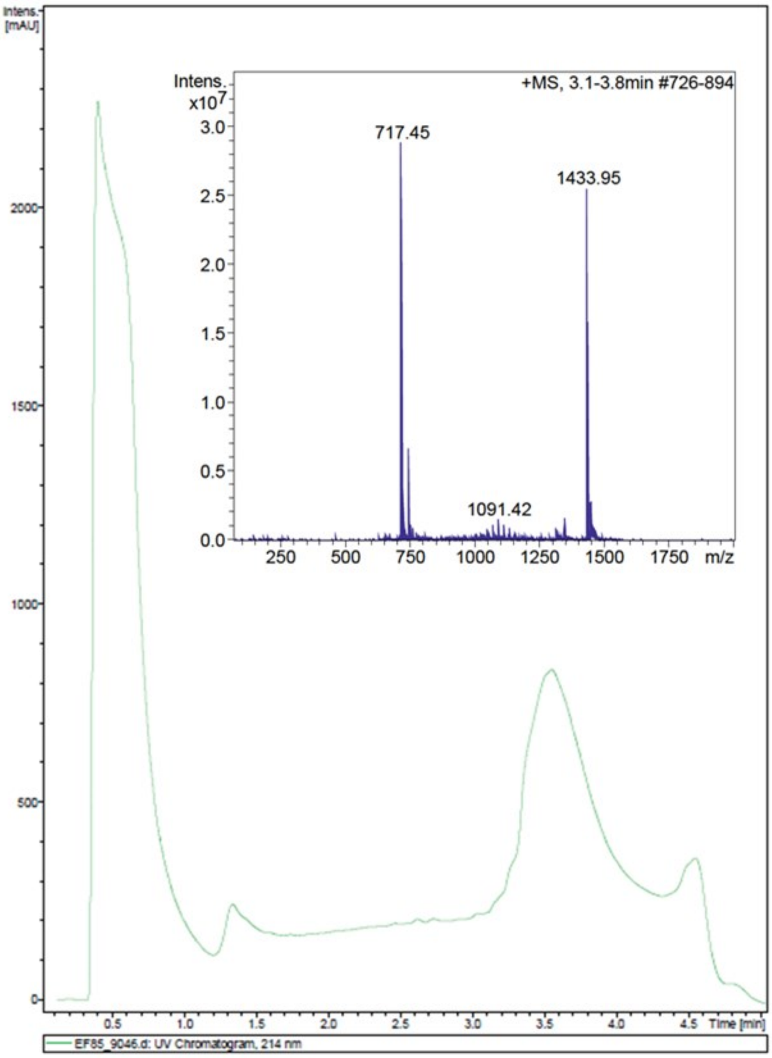

Figure S4. Mass spectrometry of the purified CP-$(XA)_2$.

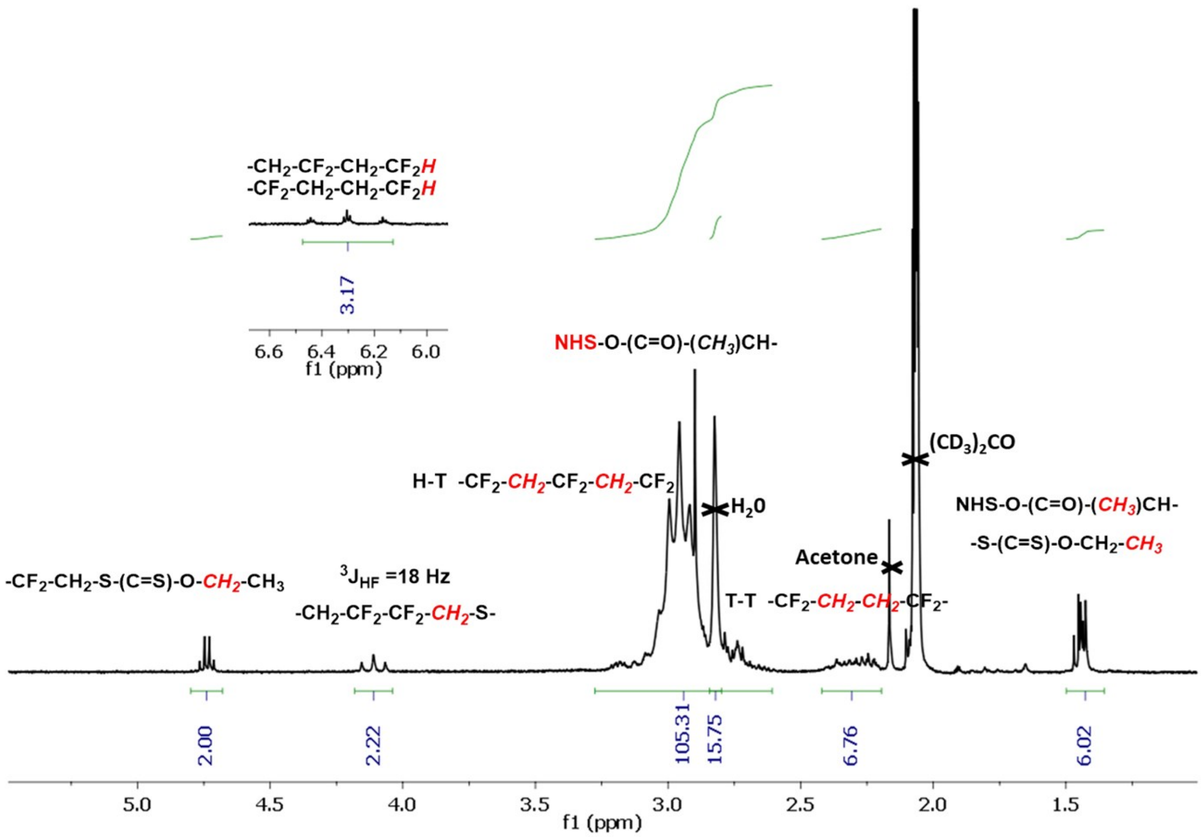

^{1}H NMR (400 MHz, $(CD_3)_2CO$, ppm, Figure S5) : δ = 1.39-1.49 (m, -CH(CH_3)(C=O)-O-NHS and –S-(C=S)-O-CH_2-CH_3), 1.65-1.85 (m, -CF_2-CH_3), $^3J_{HH}$= 7.2 Hz), 2.21-2.43 (m,-CF_2-CH_2-CH_2-CF_2-, VDF-VDF TT (tail-to-tail) reverse addition), 2.70-3.23 (t, -CF_2-CH_2-CF_2-, VDF-VDF HT (head-to-tail) regular addition), 3.60-3.69 (s, -(C=O)-O-CH_3), 4.05-4.18 (t, -CF_2-CH_2-S(C=S)OEt, $^3J_{HF}$= 18 Hz), 4.70-4.78 (q, (-S(C=S)O-CH_2-CH_3, $^3J_{HH}$= 7.1 Hz), 6.10-6.50 (tt, $^2J_{HF}$= 55 Hz , $^3J_{HH}$= 4.5 Hz -CH_2-CF_2-H).

Figure S5. ^{1}H NMR spectrum in $(CD_3)_2CO$ of a PVDF homopolymer synthesized by RAFT polymerization with NHS-CTA-XA (400 MHz)

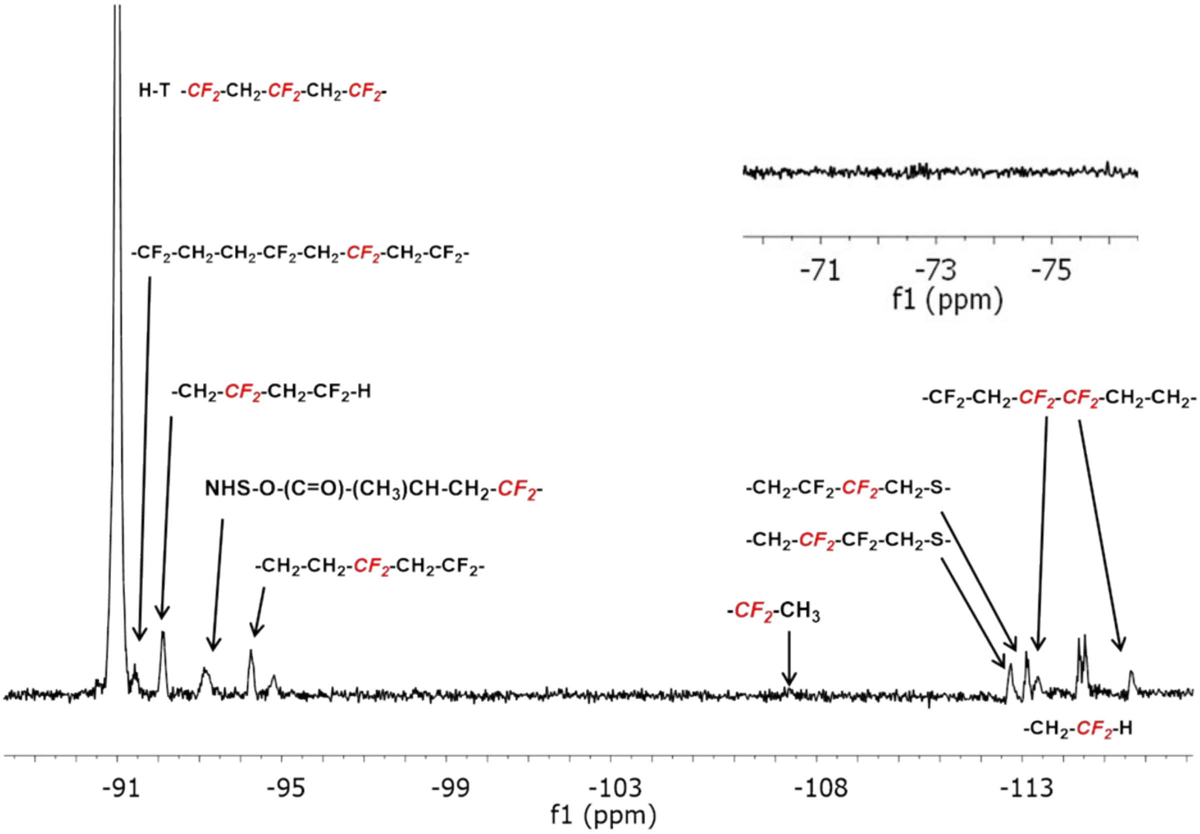

^{19}F NMR (376 MHz $(CD_3)_2CO$, ppm, Figure S6) : δ = -115.63 ($-CH_2-CF_2-CF_2-CH_2-CH_2-$, VDF-VDF HH reverse addition), -114.29 ($^2J_{HF}$= 55 Hz, $-CH_2-CF_2-H$), -113.34 ($-CH_2-CF_2-CF_2-CH_2-CH_2-$, HH reverse addition), -113.09 ($CH_2-CF_2-CF_2-CH_2-S-$), -112.69 ($-CH_2-CF_2-CF_2-CH_2-S-$), -94.79 ($-CH_2-CH_2-CF_2-CH_2-$, TT reverse addition), -107.7 ($-CF_2-CH_3$), -93.50 ($-CH_2-CF_2-CH_2-CH(CH_3)(C=O)-$), -92.12 ($-CH_2-CF_2-CH_2-CF_2H$), -91.44 ($-CH_2-CH_2-CF_2-CH_2-CF_2-CH_2-CF_2-$, regular VDF-VDF HT addition), -91.00 ($-CH_2-CF_2-CH_2-$, regular VDF-VDF HT addition).

Figure S6. ^{19}F NMR spectrum in $(CD_3)_2CO$ of a PVDF homopolymer synthesized by RAFT polymerization with NHS-CTA-XA. (376 MHz)

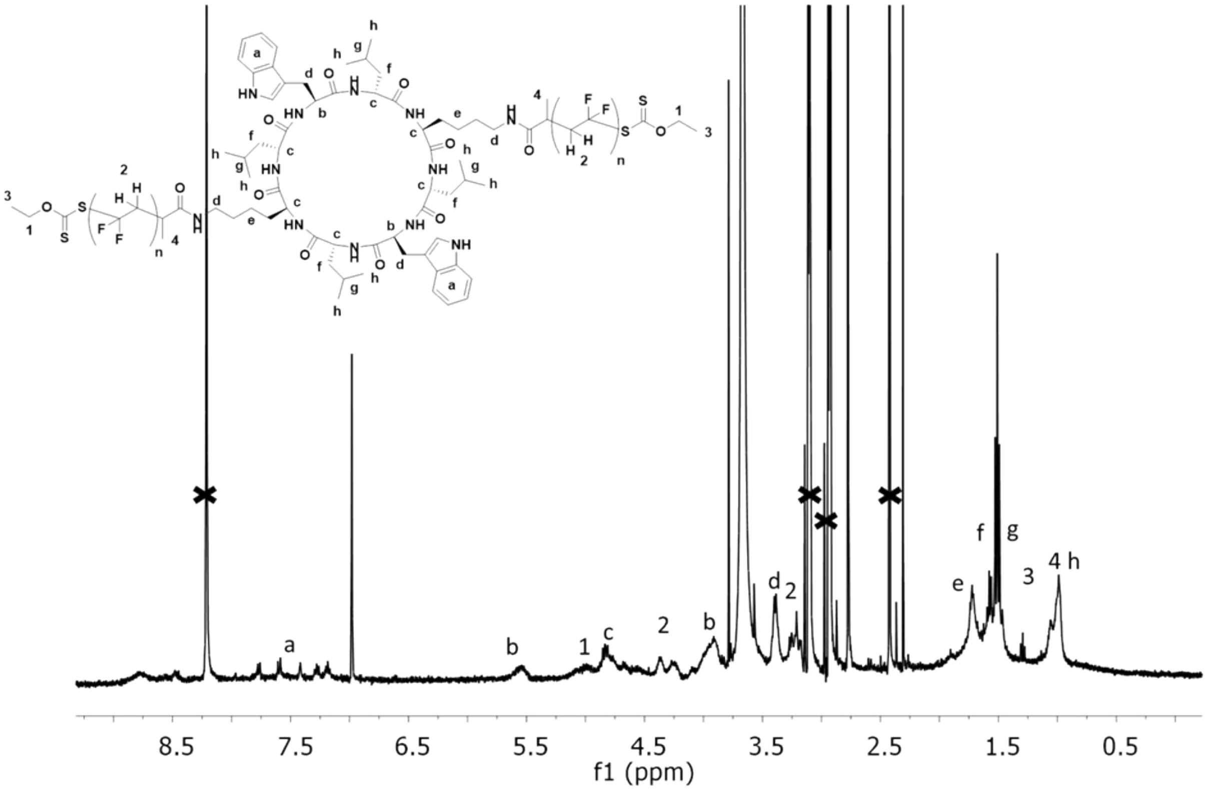

^{1}H NMR (400 MHz, C_3D_7NO, ppm, Figure S7): δ = 9.00-8.50 (m, -NH), 7.80-7.10 (m, H Trp), 5.55 (m, Hα Trp), 5.00 (m, CH_2 Z CTA), 4.85-4.50 (m, Hα Leu and Hα Lys), 4.40-4.20 (m, CF_2CH_2S-), 4.10-3.80 (m, CH_2CF_2S-), 3.40-3.30 (m, $-CH_2-$ Trp), 3.25 (t, CH_2CF_2 PVDF), 2.00-1.30 (m, CH_2-CH_2-CH_2 Lys, CH_2- CH Leu), 1.35-0.85 (m, CH_3 Leu, -CH-CH_3 R CTA, -CH_2-CH_3).

Figure S7. ^{1}H NMR spectrum in C_3D_7NO of a CP-(PVDF)$_2$ conjugate synthesized by surface initiated RAFT polymerization with CP(-XA)$_2$.

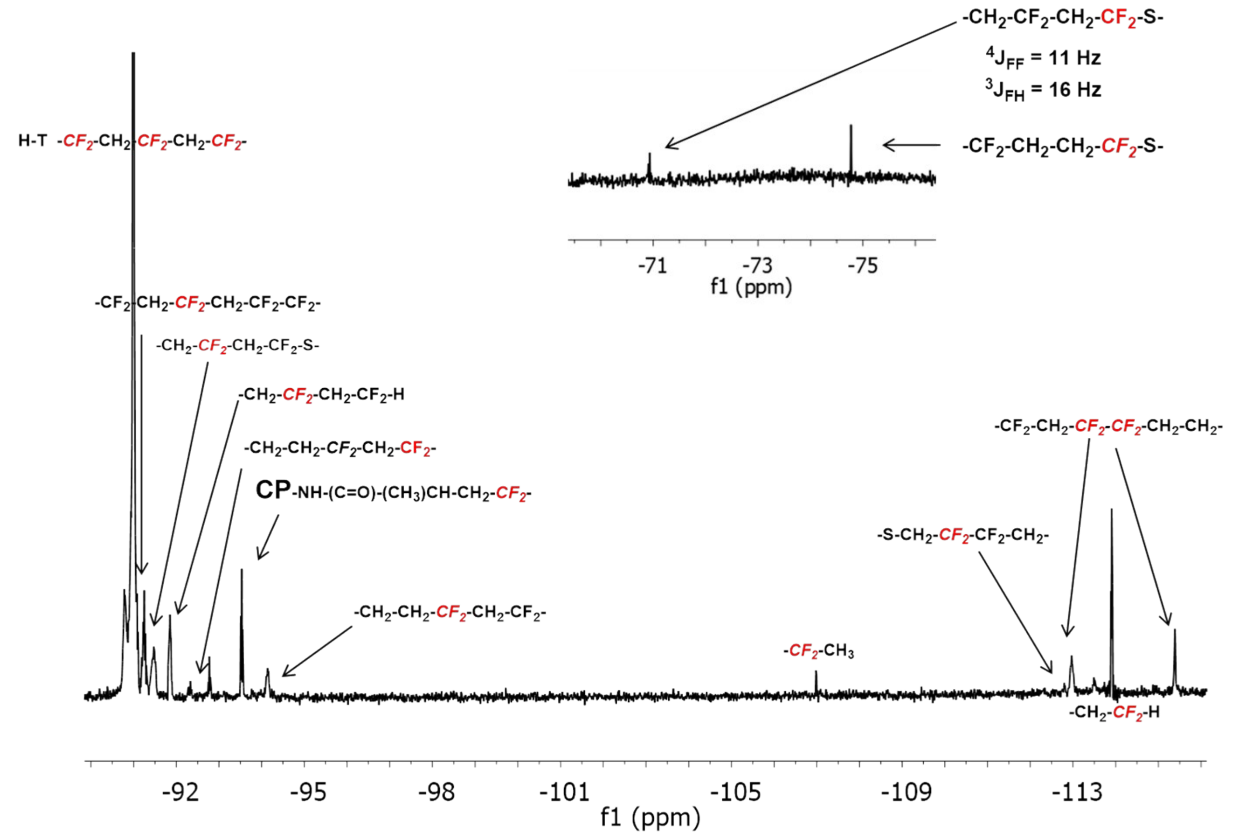

^{19}F NMR (376 MHz, C_3D_7NO, ppm, Figure S8): δ = -115.39 (-CH_2-CF_2-CF_2-CH_2-CH_2-, VDF-VDF HH reverse addition), -113.92 (-CH_2-CF_2-H), -112.97 (-CH_2-CF_2-CF_2-CH_2-CH_2-, HH reverse addition), -112.80 (-CF_2-CH_2-S-), -106.98 (-CF_2-CH_3), -94.14 (-CH_2-CH_2-CF_2-CH_2-, TT reverse addition), -93.53 (CP-NH-(C=O)-(CH_3)CH-CH_2-CF_2-), 91.85 (-CH_2-CF_2-CH_2-CF_2H), -91.45 (CH_2-CF_2-CH_2-CF_2-S-), -91.25 (-CF_2-CH_2-CF_2-CH_2-CF_2-CF_2), -91.00 (CF_2-CH_2-CF_2-CH_2-CF_2, regular VDF-VDF HT addition), -74.80 (-CF_2-CH_2-CH_2-C**F_2**-S-), -70.92 (-CF_2-CF_2-CH_2-**CF_2**-S-).

Figure S8. ^{19}F {^{1}H} NMR spectrum in C_3D_7NO of a CP-$(PVDF)_2$ conjugate synthesized by surface initiated RAFT polymerization with CP$(\text{-XA})_2$.

Table S1. M_n, M_w, M_p and Đ .

	M_n (g/mol)	M_w (g/mol)	M_p (g/mol)	*Đ*
CP	3600	5400	3500	1.49
CP-dilute	3700	7000	3300	1.86
CP-PVDF	4500	5700	4300	1.26

Theoretical DP:

$$DP_{theo} = \frac{\frac{1}{2}[VDF]_0}{[CP(XA)_2]_0} x\ Conversion = \frac{\frac{1}{2}7.81\ mmol}{6.51\ 10^{-2}\ mmol} x\ 0.5 = 30$$

(Equation S1)

DP of each arms of CP-$(PVDF)_2$ estimated from ^{19}F NMR spectrum (Figure S8) using equation S2:

$$DP = \frac{\left(\int_{-70.8}^{-71.0} + \int_{-74.7}^{-74.9} + \int_{-112.7}^{-112.9} CF_2S(\text{Z end group})\right) + \int_{-106.8}^{-107.2} CF_2CH_3 + \int_{-113.6}^{-114.1} CF_2H + \left(\int_{-90.9}^{-94.0} + \int_{-112.8}^{-113.2} + \int_{-115.2}^{-115.8} CF_2(\text{(HT), (HH), (R end group) and (H termination)})\right)}{\left(\int_{-70.8}^{-71.0} + \int_{-74.7}^{-74.9} + \int_{-112.7}^{-112.9} CF_2S(\text{Z end group})\right) + \int_{-106.8}^{-107.2} CF_2CH_3 + \int_{-113.6}^{-114.1} CF_2H}$$

(Equation S2)

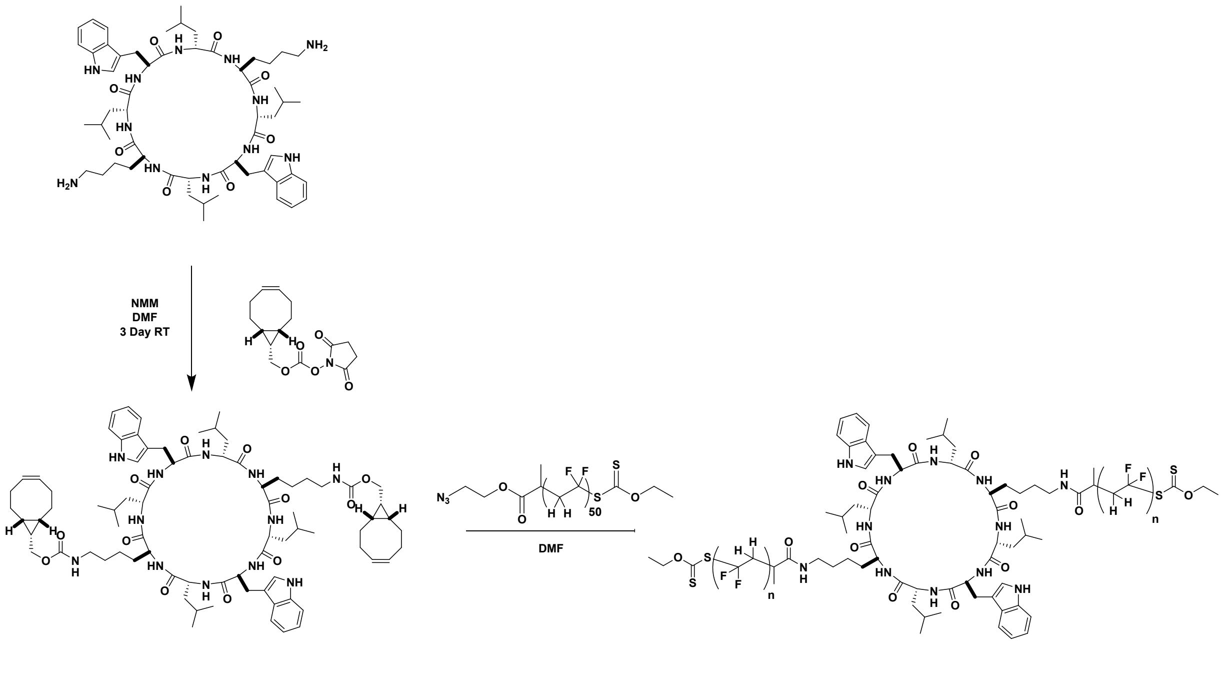

Figure S9. Synthetic route to the cyclic peptide-PVDF conjugate employing a »grafting-to » approach.

$$(S3)\ (\%) - CF_2 - CH_2 - XA = \frac{\int_{-112.85}^{-112.72} - CF_2 - CH_2 - XA}{\int_{-107.02}^{-106.95} - CF_2 - CH_3 + \int_{-74.82}^{-74.70} CH_2 - CH_2 - CF_2 - XA + \int_{-113.95}^{-113.85} - CH_2 - CF_2H + \int_{-112.85}^{-112.72} - CF_2 - CH_2 - XA}$$

$$(S4)\ (\%) - CH_2 - CF_2H = \frac{\int_{-113.95}^{-113.85} - CH_2 - CF_2H}{\int_{-107.02}^{-106.95} - CF_2 - CH_3 + \int_{-74.82}^{-74.70} CH_2 - CH_2 - CF_2 - XA + \int_{-113.95}^{-113.85} - CH_2 - CF_2H + \int_{-112.85}^{-112.72} - CF_2 - CH_2 - XA}$$

$$(S5)\ (\%) - CH_2 - CF_2 - XA = \frac{\int_{-74.82}^{-74.70} CH_2 - CH_2 - CF_2 - XA}{\int_{-107.02}^{-106.95} - CF_2 - CH_3 + \int_{-74.82}^{-74.70} CH_2 - CH_2 - CF_2 - XA + \int_{-113.95}^{-113.85} - CH_2 - CF_2H + \int_{-112.85}^{-112.72} - CF_2 - CH_2 - XA}$$

$$(S6)\ (\%) - CF_2 - CH_3 = \frac{\int_{-107.02}^{-106.95} - CF_2 - CH_3}{\int_{-107.02}^{-106.95} - CF_2 - CH_3 + \int_{-74.82}^{-74.70} CH_2 - CH_2 - CF_2 - XA + \int_{-113.95}^{-113.85} - CH_2 - CF_2H + \int_{-112.85}^{-112.72} - CF_2 - CH_2 - XA}$$

Equations used to calculate the proportions of chain-ends. Chain-end proportions were calculated using data from ^{19}F NMR of CP(PVDF)$_2$ (Fig S8).[1]

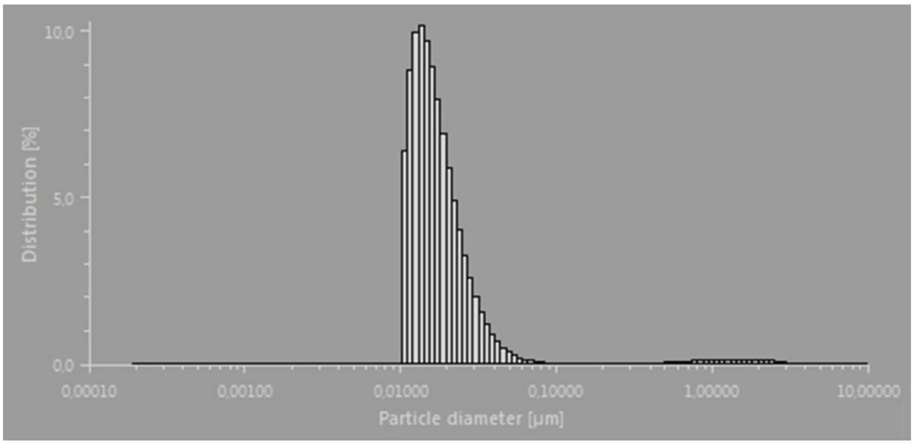

Figure S10. DLS number-average hydrodynamic diameter distribution of CP(XA)$_2$ conjugates (13.3 mg mL^{-1}) in acetone after heating at 50 °C (20 min) and sonication (20 min).

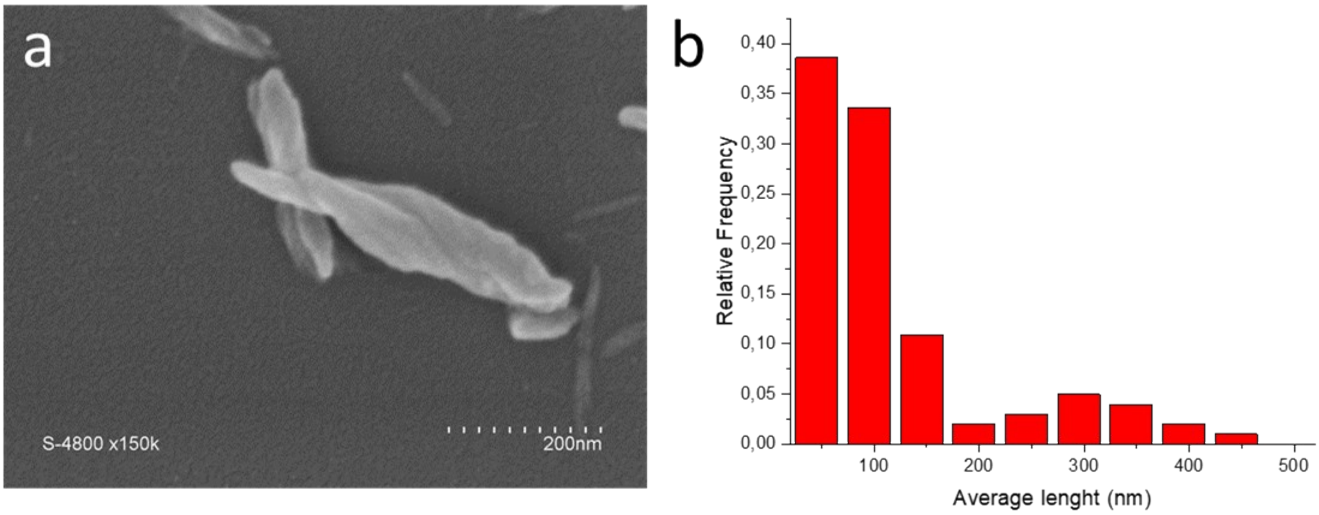

Figure S11. a) SEM image of twisted ribbon structures observed in the diluted suspension shown in Figure 2a. b) size distribution of the assemblies observed in Figure 2b.

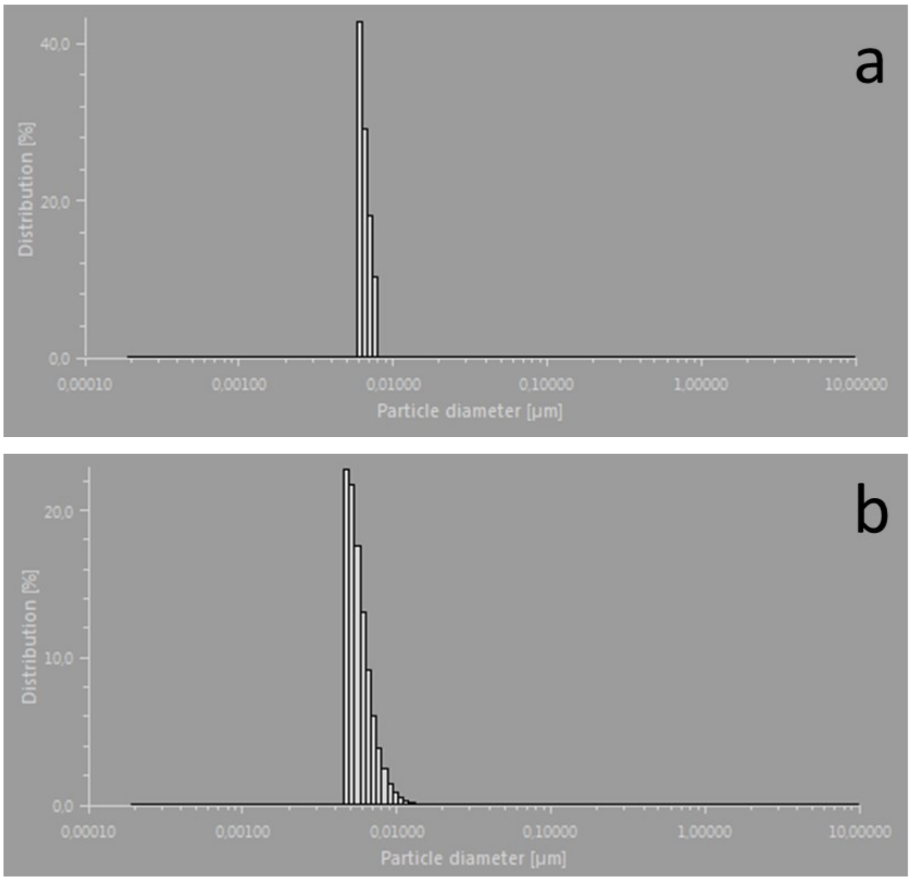

Figure S12. DLS intensity-average hydrodynamic diameter distribution of CP(PVDF)$_2$ conjugates (1 mg mL^{-1}) in (a) DMF after heating at 60 °C (1h) and sonication (20 min) and (b) DMSO after heating at 80 °C (1h) and sonication (20 min).

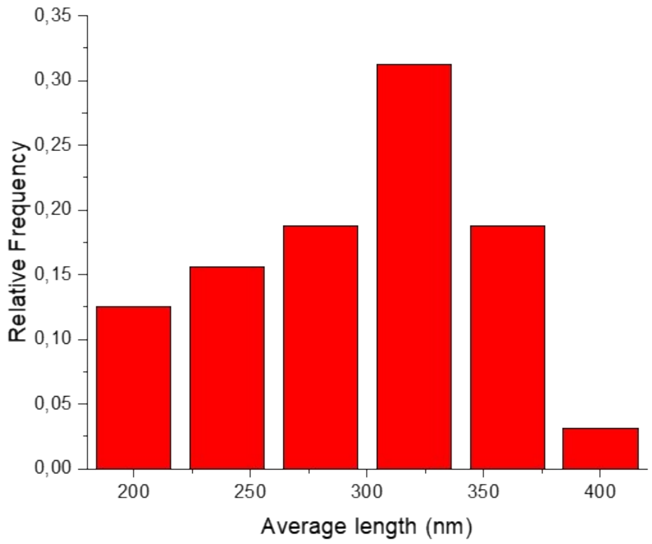

Figure S13. Size distribution of the assemblies observed in Figure 4c and 4d
